# Task-Specific Perceived Harmfulness Predicts Protective Movement Behaviour in Chronic Low Back Pain

**DOI:** 10.3390/jcm13175025

**Published:** 2024-08-25

**Authors:** Thomas Matheve, Annick Timmermans, Lieven Danneels, Liesbet De Baets

**Affiliations:** 1 Spine, Head and Pain Research Unit Ghent, Department of Rehabilitation Sciences, Ghent University, 9000 Gent, Belgium; lieven.danneels@ugent.be; 2 REVAL—Rehabilitation Research Center, Faculty of Rehabilitation Sciences, UHasselt, 3500 Diepenbeek, Belgium; annick.timmermans@uhasselt.be; 3 Pain in Motion Research Group (PAIN), Department of Physiotherapy, Human Physiology and Anatomy, Faculty of Physical Education and Physiotherapy, Vrije Universiteit Brussels, 1050 Brussels, Belgium; liesbet.debaets@kuleuven.be; 4 Musculoskeletal Rehabilitation Research Group, Department of Rehabilitation Sciences, Faculty of Movement and Rehabilitation Sciences, KU Leuven, 3001 Leuven, Belgium

**Keywords:** low back pain, fear of movement, movement behaviour, movement velocity, kinematics

## Abstract

**Background/Objectives:** There is emerging evidence that task-specific pain-related psychological measures may better predict movement behaviour in chronic low back pain (CLBP) than general pain-related psychological measures. Currently, little is known regarding the prediction of movement duration and movement velocity. **Methods**: Baseline data from a previously published randomized controlled trial were used (clinicaltrials.gov NCT02773160). Fifty-five patients with CLBP and 54 pain-free persons performed a lifting task while kinematic measurements were obtained to calculate movement velocity of the L1 vertebra, S1 vertebra, and the lumbar spine, as well as the time to complete the lifting task. Scores on the Photograph Daily Activities Series-Short Electronic Version (PHODA-SeV), Tampa Scale for Kinesiophobia (TSK), and its Activity Avoidance and Somatic Focus subscales were used as general pain-related psychological measures. The score on a picture of the PHODA-SeV, showing a person lifting an object with a bent back (PHODA-Lift), was used as task-specific measure of perceived harmfulness. **Results**: The task-specific measure best predicted movement duration and movement velocity of L1 and the lumbar spine, and explained 35%, 19%, and 25% of the respective movement parameters. Although general perceived harmfulness predicted S1 velocity and movement duration, it only explained 6% and 8% of the respective movement parameters. General measures of pain-related fear were not predictive for any of the movement parameters. It took patients with CLBP significantly longer to complete the lifting task when compared to the pain-free participants (ES = 1.01, *p* < 0.0001), and patients with CLBP also moved significantly slower at L1 (ES = 0.85, *p* < 0.0001) and the lumbar spine (ES = 1.01, *p* < 0.0001). These between-groups differences were larger for CLBP subgroups with higher scores on the PHODA-Lift, and to some extent for subgroups with higher total scores on the PHODA-SeV. **Conclusions**: Task-specific perceived harmfulness best predicts movement velocity. General pain-related fear measures (i.e., TSK and its subscales) do not predict these movement parameters.

## 1. Introduction

Low back pain (LBP) has been identified as the leading cause of years lived with disability worldwide [1]. Especially in the chronic stage, LBP becomes notoriously difficult to treat [2]. One of the main drivers for the development and persistence of chronic low back pain (CLBP) is fear of movement and avoidance behaviour [3]. While avoiding painful movements in acute pain can be adaptive—as it unloads tissues, allowing them to heal—avoidance behaviour becomes unhelpful when it persists after tissues have healed and protection is no longer necessary [4]. As a result, this avoidance behaviour will interfere with valued life activities and lead to chronic pain-related disability [5]. This is highly relevant in chronic low back pain (CLBP) as no pathoanathomical cause for the pain can be identified in about 90% of this population, so continued tissue protection is unnecessary [6].

Upon confrontation with a threatening situation, defensive activation can occur in various systems. For example, certain cognitions (e.g., catastrophizing thoughts), psychophysiological responses (e.g., changes in skin conductance), and behavioural responses (e.g., avoidance) may be present (See reviews for more details on this topic [7,8]). Regarding the latter, the well-established fear-avoidance model of pain posits that unhelpful avoidance behaviour develops and persists when people hold negative beliefs about pain [5]. More specifically, perceiving pain as highly threatening and as a sign of bodily harm—even when tissues have healed—will lead to fear of movement and subsequent avoidance behaviour [5,9]. However, while fear of movement and avoidance behaviour are traditionally considered to be strongly related to each other [10], various recent systematic reviews have only shown weak associations between self-reported fear of movement and actual movement behaviour in CLBP [11,12,13,14]. An important reason is that most of the previous studies investigating these associations have only used general self-report measures assessing fear of movement (e.g., Tampa Scale for Kinesiophobia) [15]. These general measures typically contain items that only pertain to physical activity or exercises in general, thereby ignoring that many people with CLBP only fear and avoid specific activities (e.g., lifting with a bent back) without displaying a generalised fear of movement [15]. 

As an alternative, using task-specific measures may be more appropriate to assess the relationships between pain-related psychological measures and movement behaviour [15]. Recent studies have indeed shown that such task-specific measures better predict movement behaviour in patients with CLBP [16,17,18] and even in pain-free participants [19,20]. However, only one of these studies has investigated movement velocity [17]. Yet, there is emerging evidence that movement velocity is an important parameter to characterize avoidant movement behaviour in CLBP [21,22] as changes in movement velocity—typically, an increase—have been related to reduced pain and disability after treatment [22,23]. Therefore, investigating whether task-specific pain-related psychological measures better predict movement velocity than general measures is important to further disentangle the complex relationships between pain, psychological measures, and movement behaviour in patients with CLBP.

As such, the aims of this study were (1) to investigate whether movement duration and movement velocity are related to general or task-specific pain-related psychological measures in patients with CLBP, (2) to investigate whether movement duration and movement velocity differ between patients with CLBP and pain-free persons, and (3) to evaluate whether these potential between-groups differences depend on the scores on the pain-related psychological measures in the CLBP group. 

## 2. Materials and Methods

### 2.1. Design

This cross-sectional study is based on baseline data from a published randomized controlled trial [24] (clinicaltrials.gov NCT02773160). We previously reported results of the relationships between pain-related psychological measures and lumbar range of motion [16]. Here, we present data regarding movement velocity and duration.

### 2.2. Participants

Fifty-four pain-free persons and 55 patients with chronic nonspecific LBP participated in this study. Participants were recruited via private general practitioner and physiotherapy practices and via social media. To be included, participants needed to be between 18 and 65 years old, and patients had to be diagnosed with chronic non-specific LBP. The criterion “chronic” was defined as LBP lasting longer than 3 months, during which patients experienced LBP on at least 3 days per week, while the term non-specific indicates that the CLBP cannot (confidently) be attributed to a specific pathoanatomic cause or underlying disease [6,25]. Exclusion criteria for both participant groups were signs and symptoms of nerve root involvement, a serious underlying disease, axial spondylarthropathy, fibromyalgia, pregnancy, performance of lumbopelvic movement control exercises in the past year, and musculoskeletal complaints other than LBP interfering with daily functioning (e.g., severe knee pain). Pain-free participants were excluded when they experienced at least 1 day of self-reported LBP in the past year or when they sought professional help for their LBP in the past year. Persons willing to participate in the study were initially screened via a structured telephone interview, and eligibility was confirmed when participants arrived in the laboratory, prior to the start of the tests. This was done via a patient interview and clinical examination when necessary. When an exclusion criterion was suspected or present (e.g., nerve root involvement), participants were excluded. No medical imaging (e.g., MRI) was performed to make this decision. Ethical approval was obtained from the Ethics Committees of Hasselt University and Jessa Hospital, Belgium (approval number B243201423040). All participants gave written informed consent before being included in the study.

### 2.3. Procedures

After eligibility confirmation in the lab, participants first completed a set of questionnaires. Hereafter, participants were prepared for the kinematic measurements (e.g., mounting of inertial measurement units), after which they performed the lifting task.

#### 2.3.1. Questionnaires

Pain-free participants and patients with CLBP:

- *Sociodemographic data*: Age and sex were collected via self-report, while height and weight were measured at the lab.

**Figure 1:**
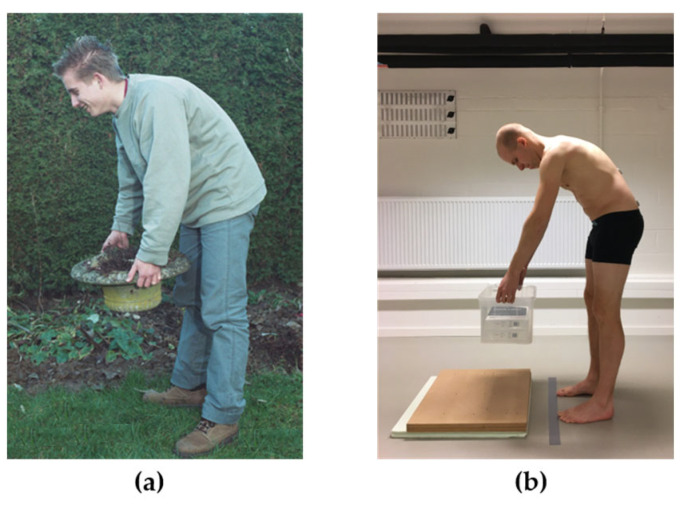
(**a**) Task-specific measure of perceived harmfulness (PHODA-Lift); (**b**) Standardised lifting task performed by the participants. For clarity, participants were instructed to lift in their habitual way, so they were also allowed to bend their knees or keep their back straight. The lifting strategy in (**b**) does not necessarily represent the strategy that participants used to lift the box.

Patients with CLBP:

- *Numeric Pain Rating Scale (NPRS)* [23]: Both the current LBP intensity and the average LBP intensity over the past 7 days were collected on a 0 to 10 numeric rating scale (0 = no pain, 10 = worst imaginable pain).
- *LBP duration:* Participants were asked to indicate the duration of their current episode of CLBP.
- *Roland Morris Disability Questionnaire (RMDQ)* [24]: The RMDQ assesses LBP-related disability and consists of 24 items that have to be answered with yes or no. A higher score (range 0–24) represents a higher level of LBP-related disability.
- *Tampa Scale for Kinesiophobia (TSK)* [25]: The TSK is a questionnaire containing 17 items to assess subjective ratings of fear of movement/re-injury due to physical activity. The total score (TSK-total) ranges between 17 and 68, with a higher score indicating a higher level of pain-related fear. For patients with CLBP, two subscales can be discerned in the TSK. The Activity Avoidance subscale (TSK-AA) specifically measures activity avoidance and fear of re-injury, whereas the Somatic Focus subscale (TSK-SF) assesses to which extent patients believe that their LBP can be attributed to a serious underlying medical problem [26]. Because it has previously been hypothesized that the TSK-AA might stronger associated with movement-related parameters than the TSK-total or TSK-SF [27], a separate score for the TSK-AA (range 8–32) and for the TSK-SF (range 5–20) was also calculated.
- *The Photograph Series of Daily Activities—Short Electronic Version (PHODA-SeV)* [28]: The PHODA-SeV assesses the perceived harmfulness of various daily life activities. Participants are shown pictures of persons performing daily life activities (40 in total), and they are asked to imagine themselves performing these activities as shown on the pictures. For each activity, participants need to indicate to which extent they think the activities are harmful to their back, by using a 0 to 100 scale (0 = not harmful at all, 100 = extremely harmful). A total score (0 to 100) is calculated by averaging the scores of the 40 pictures (=PHODA-Total). The score (range 0 to 100) on one specific picture of the PHODA-SeV, displaying a lifting task with a bent back (see Figure 1a), was defined a priori as a task-specific measure of perceived harmfulness (=PHODA-lift), as this task is very similar the task that participants had to perform in the current study (i.e., lifting an object of similar dimensions).

The total score on the PHODA-SeV (=PHODA-Total) provides a general indication of perceived harmfulness as it represents an average of all the individual scores of the 40 tasks. Therefore, the score on the PHODA-Total is considered a general measure of perceived harmfulness. In a similar vein, the TSK and its subscales do not contain items that specifically relate to lifting objects. As such, the TSK-Total, TSK-AA, and TSK-SF are considered general measures of pain-related fear. 

#### 2.3.2. Movement Task

The movement task started with the participants standing in their habitual position. Next, they were instructed to lift a box from a platform, to remain in an upright standing position for 1 s after lifting the box, after which they had to place the box on the platform again (See Figure 1b). Participants were instructed to lift the box in their habitual way (“lift the box in your habitual way, as you would do at home”). The task was performed five times at a self-selected pace. To standardize the lifting task for the participants’ height, the top of the box was positioned 10 cm below the apex of the subjects’ patella. Participants stood with both feet at a distance of 15 cm from the box. The dimensions of the box were 40 × 30 × 23.5 cm, and it weighed 4 kg. The dimensions and weight of the box were chosen as they closely resemble the object in the picture of the PHODA-lift. Furthermore, we used this particular task because lifting is a highly relevant activity that patients with CLBP typically perceive as harmful [28], and lumbopelvic kinematics during this task can be assessed with excellent reliability [29,30].

#### 2.3.3. Kinematic Data Acquisition and Processing

The Valedo^®^motion research tool (version 1.2; Hocoma, Volketswil, Switzerland) was used for the kinematic measurements. This tool consists of wireless inertial measurement units (IMUs) that have a sampling rate of 50 Hz and measure with a 0.1° accuracy, and the tool is reliable and valid for assessing lumbopelvic kinematics [29,30]. The measurements started by palpating the lateral condyle of the left femur and the spinous processes of L1 and S1 in a standardized way [31]. Next, the system was calibrated and the IMUs were mounted 20 cm above the femoral condyle and on the spinous processes of L1 and S1 using double-sided tape. These procedures were performed with the participants standing in their habitual way. The kinematic data acquisition started by recording the habitual standing position of the participants. 

Movement velocity during the lifting phase was calculated using the three middle repetitions (i.e., repetition 2, 3, and 4) to eliminate potential influence of movement initiation and conclusion during the respective first and last movement cycle. To calculate movement velocity during the lifting phase, we first determined movement cycles visually. The start of a movement cycle was determined by movement initiation of any of the three IMUs towards an upwards movement direction in the sagittal plane (i.e., lifting the box). The end of a movement cycle was determined when the person stood upright with the box in their hands, and all three IMUs remained stationary. In some cases, participants moved their lumbar spine or hip joint slightly into hyperextension, after which slight flexion occurred towards the starting position. In these cases, the end of the movement cycle was determined at the end of the extension movement. To calculate movement velocity, we first determined the angular displacement (°) of the L1 and S1 IMUs during each of the three movement cycles. In addition, range of motion of the lumbar spine was calculated using the angular displacements of the L1 IMU relative to the S1 IMU. The velocity of L1, S1, and the lumbar spine were calculated for each of the three repetitions by dividing the angular displacements by the time of the movement cycle (=movement duration), resulting in movement velocity expressed as degrees per second (°/s). For clarity, we only used data from the lifting-up phase—not from the putting-down phase—as participants typically interpret the PHODA-Lift picture as lifting an object with a bent back, and not as putting it down [13]. The reason for calculating movement velocity of L1 and S1—in addition to lumbar spine movement velocity—is that this may provide different information. For example, in persons with a dominant hip movement pattern (i.e., bending forward in the hips while keeping a “straight” back), there will be a relatively similar angular displacement of L1 and S1, with little movement at the lumbar spine itself. In this case, angular movement of L1 and S1 will be much larger than lumbar movement, leading to much higher movement velocities at L1 and S1 when compared to the lumbar spine. In addition, changes in movement behaviour (including movement velocity) after rehabilitation have been shown to be larger at L1 than at the lumbar spine [19].

### 2.4. Statistical Analyses

To assess the relationships between the pain-related psychological measures and movement velocity, we first calculated the correlation coefficients between the scores on the TSK-Total, TSK-AA, TSK-SF, PHODA-Total, and PHODA-Lift on the one hand, and movement velocity of L1, S1, and the lumbar spine on the other hand. In addition, we calculated the correlation coefficients between the pain-related psychological measures and the duration of the lifting task (expressed in seconds). Pearson correlation coefficients were used when data were normally distributed, and Spearman correlation coefficients were used when this was not the case. Correlation coefficients were interpreted as small (r < 0.30), medium (0.30 ≤ r < 0.50) and large (r ≥ 0.50) [26]. Next, we performed multiple linear regressions. First, a basic model was constructed that only contained the controlling variables age, sex, current pain, duration of the current CLBP episode, and disability. These variables were controlled for as they might influence lumbopelvic movement behaviour in patients with LBP [27,28,29,30]. Next, for every pain-related psychological measure (i.e., TSK-total, TSK-AA, TSK-SF, PHODA-total, PHODA-lift), a separate regression analysis was made by adding the respective measure to the basic model. This resulted in five additional regression models per movement parameter, each containing the same set of controlling variables, but a different pain-related psychological measure. The movement parameters (movement velocity of L1, S1, lumbar spine, and movement duration) were the dependent variables of the multiple linear regression analyses. 

To compare movement velocity and duration between pain-free persons and patients with CLBP, we performed multiple linear regression analyses using age and sex as controlling variables. First, pain-free participants were compared with the CLBP group as a whole. Second, to assess whether pain-related psychological measures influenced this comparison, patients with CLBP were categorized into subgroups of low, medium, or high scores on the respective pain-related psychological measures by using the lowermost, middle, and uppermost thirds of the actual scores [31]. This subgroup analysis was performed for each of the pain-related psychological measures. Dunnett’s post hoc test was used to compare each subgroup of patients (low, middle, or high level) with the group of pain-free persons. Between-group effect sizes were calculated using the mean estimates of the regression analyses. When comparing the pain-free group with the complete CLBP group, Cohen’s d was calculated; for comparison with CLBP subgroups, we calculated Hedges’ g because of the smaller sample sizes in the CLBP subgroups. Effect sizes were interpreted as small (0.20), medium (0.50), large (0.80), and very large (>1.2) [26].

### 2.5. Sample Size

Since data for this study are from baseline measurements of an RCT, our original sample size was determined for our previously published RCT. The main outcome of our study is the R^2^ increase by adding the respective pain-related psychological measure to the basic model. Based on our previous study [16] and the study by Imai et al. [17], it can be expected that the R^2^ of the basic model would be at least 0.35. Taking into account that we have five predictors in the basic model, and that we are interested in the R^2^ increase by adding one predictor variable to the basic model, we would be able to detect a significant R^2^ increase of 0.10, considering α = 0.05 and a power of 0.8. These calculations were performed using G*Power (version 3.1.9.7). 

## 3. Results

### 3.1. Participants

**Figure 2:**
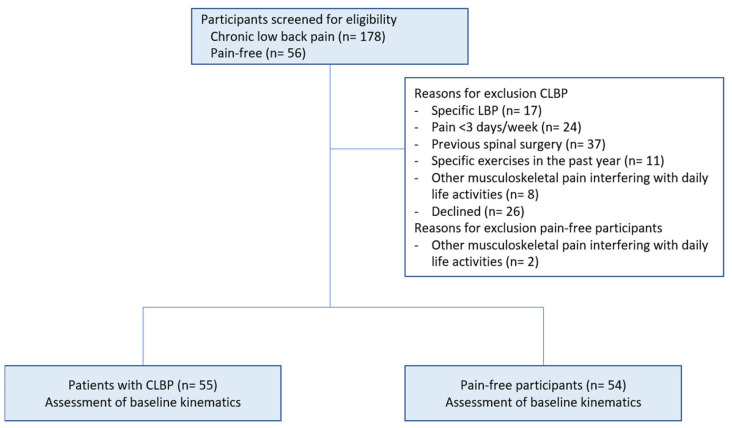
Flowchart of the study with reasons for exclusion.

We screened 178 patients with CLBP and 56 pain-free participants, and we included 55 patients with CLBP and 54 pain-free participants. Reasons for exclusion can be found in Figure 2. Sociodemographic data and scores on the questionnaires can be found in Table 1.

### 3.2. Relationships between Pain-Related Psychological Measures and Movement Parameters

Table 2 shows the correlation coefficients between the pain-related psychological measures and movement velocity and duration. Overall, the correlation coefficients between the movement parameters and general pain-related psychological measures (TSK-Total, TSK-AA, TSK-SF, and PHODA-Total) were small and statistically nonsignificant, except for the moderately positive correlation coefficient between the PHODA-Total and movement duration (r = 0.37, *p* = 0.007), indicating that participants with CLBP and higher PHODA-Total scores took longer to complete the lifting task. Strong correlation coefficients were found between the task-specific measure of perceived harmfulness (PHODA-Lift) and movement duration (r = 0.63, *p* < 0.0001), and the movement velocity of L1 (r = −0.55, *p* < 0.0001) and the lumbar spine (r = −0.57, *p* < 0.0001). This indicates that people with higher scores on the PHODA-Lift took longer to complete the lifting task and moved significantly slower at L1 and at the lumbar spine.

Table 3 and Table 4 present the multiple linear regression models predicting movement velocity and movement duration. Only the regression models including the PHODA-Total and PHODA-Lift are shown, as none of the general measures of pain-related fear (i.e., TSK-Total, TSK-AA, and TSK-SF) significantly predicted movement velocity or duration. For the regression models including the general measures of pain-related fear, we refer to Supplementary Tables S1–S3. The general measure of perceived harmfulness (PHODA-Total) was a significant predictor of movement duration when added to the basic model (*p* = 0.02). Since the PHODA-Lift was also a significant predictor of movement duration, we constructed a regression model including the control variables and both the PHODA-Total and PHODA-Lift. In this model, the PHODA-Total became statistically non-significant (*p* = 0.91), whereas the PHODA-Lift retained its statistical significance (*p* < 0.0001). The PHODA-Total was significant in the regression model predicting S1-velocity, where it explained an additional 6% in the variance (*p* = 0.04). The task-specific measure of perceived harmfulness (PHODA-Lift) remained statistically significant for lumbar spine velocity, L1 velocity, and movement duration. The PHODA-Lift explained an additional variance (ΔR^2^ adj) to the basic model of 0.25 for lumbar spine velocity (*p* < 0.0001), 0.19 for L1 velocity (*p* = 0.0006), and 0.35 for movement duration (*p* < 0.0001).

### 3.3. Comparison between Patients with Chronic Low Back Pain and Pain-Free Persons

#### 3.3.1. Complete Chronic Low Back Pain Group vs. Pain-Free Participants

When comparing the complete CLBP group to the pain-free group, patients with CLBP moved significantly slower at the lumbar spine (d = 1.02; *p* < 0.0001) and at L1 (d = 0.85; *p* < 0.0001), and it took them significantly longer to complete the lifting task (d = 1.01; *p* < 0.0001). No between-groups differences were found for S1 velocity (d = 0.17; *p* = 0.37). Details can be found in Table 5.

#### 3.3.2. Chronic Low Back Pain Subgroups vs. Pain-Free Participants

Differences in movement parameters between pain-free participants and patients with CLBP were not dependent on the levels of general pain-related fear (TSK-Total, TSK-AA, and TSK-SF) (see Supplementary Tables S4–S6 for details). All the differences in movement parameters between the pain-free participants and any of the subgroups with low, medium, or high scores on the general pain-related fear measures were similar to one another and to those between the pain-free and the complete CLBP groups. Overall, effect sizes were large and significant for lumbar spine and L1 velocity, and also for movement duration (Hedges’ g range = 0.64 to 1.15; all *p*-values ≤ 0.03). Small and non-significant effect sizes were present for S1 velocity (Hedges’ g range = 0.08 to 0.23; all *p*-values ≥ 0.76). Different levels of task-specific perceived harmfulness (PHODA-Lift) clearly influenced between groups-differences for lumbar spine and L1 velocity, and also for movement duration (Table 6). Non-significant small to moderate effect sizes were present between pain-free participants and the subgroup with low levels of task-specific perceived harmfulness (Hedges’ g range = 0.26 to 0.5; all *p*-values ≥ 0.2), while effect sizes were significant and large for the subgroup with medium levels (Hedges’ g range = 0.7 to 1.13) and very large for the subgroup with high levels of task-specific perceived harmfulness (Hedges’ g range = 1.58 to 2.12). Differences in L1 velocity and movement duration between pain-free participants and patients with CLBP were also dependent on the levels of general perceived harmfulness (PHODA-Total) (Table 7). These differences were moderate and non-significant for the subgroup with low levels of general perceived harmfulness (Hedges’ g range = 0.53 to 0.54; all *p*-values ≥ 0.11), while they were significant and large for the medium subgroup (Hedges’ g range = 0.83 to 0.86; all *p*-values ≤ 0.006) and large to very large for the high subgroup (Hedges’ g range = 1.15 to 1.34; all *p*-values ≤ 0.006).

## 4. Discussion

We investigated the relationships between pain-related psychological measures and movement duration as well as movement velocity during a lifting task, while controlling for various parameters that may influence lumbar movement behaviour. We showed that movement velocity of L1 and the lumbar spine, and also movement duration, was predicted by a task-specific measure of perceived harmfulness (PHODA-Lift), which explained an additional variance of 19%, 25%, and 35% in the respective movement parameters. A general measure of perceived harmfulness (PHODA-Total) predicted S1 velocity, although it only explained an additional 6% of the variance in this movement parameter. Regarding between groups-differences, it took the patients with CLBP significantly longer to complete the lifting task when compared to the pain-free participants, and patients with CLBP also moved significantly slower at L1 and the lumbar spine. These between-groups differences were larger for CLBP subgroups with higher scores on the PHODA-Lift, and to some extent for subgroups with higher PHODA-Total scores. General measures of pain-related fear (TSK-Total, TSK-AA, and TSK-SF) were not related to any of the movement parameters, nor was the magnitude of the between-groups differences dependent on the scores on these self-reported measures.

We previously reported results on lumbar range of motion that were based on the same dataset that we used for the current study [16]. The correlation coefficients (range absolute values r = 0.55–0.63) between the task-specific perceived harmfulness and three of four movement parameters examined in the current study were larger than the correlation coefficient we previously found between task-specific perceived harmfulness and maximal range of motion in the lumbar spine (absolute value r = 0.43) [16]. In a similar vein, the additional variance explained by the task-specific measure of perceived harmfulness for these parameters was larger for movement velocity and duration (range = 19–35%) than for range of motion (11%) [16].

Patients with CLBP moved significantly slower at the lumbar spine and at the L1-level, and it took them longer to complete the lifting task when compared to the pain-free participants. The observed differences with the pain-free group were particularly large for the CLBP subgroups with higher levels of task-specific perceived harmfulness, and to a lesser extent for the subgroups with elevated levels of general perceived harmfulness. This confirms previous research, showing that reduced movement velocity in (chronic) LBP populations is present [29], but also depends on CLBP-subgroups [32]. For example, Kent et al. [32] showed that movement duration was longest, and velocity was slowest, in a subgroup of CLBP with the least lumbopelvic range of motion during forward bending. In addition, Kent et al. also reported that 25% of CLBP participants had a movement pattern similar to that of the majority of pain-free participants. In this respect, it is interesting that differences in all movement parameters between the subgroup of CLBP patients with low levels of task-specific perceived harmfulness and the pain-free participants were not statistically significant in our study. This provides further evidence for the heterogeneity of the CLBP population, where a subgroup of patients appears to exhibit similar movement patterns to those of pain-free participants [21,32,33,34].

One previous study investigated the relationships between a task-specific pain-related fear measure and lumbar movement velocity in CLBP [17]. In line with results from our current study, Imai et al. [17] showed that task-specific but not general measures of pain-related fear were associated with peak movement velocity of the lumbar spine during forward bending. However, task-specific fear was assessed after participants completed the movement task, by asking to indicate on a 0–10 scale, “how much fear of movement they experienced during the movement task”. Assessing self-reported fear immediately after task-performance may significantly affect the self-report—as participants are likely to be influenced by the movement experience itself—potentially inflating relationships between the self-report and the movement behaviour [7]. In our study, participants first completed the PHODA-SeV, which contained 40 pictures that had to be assessed for perceived harmfulness. The task-specific measure was one of these pictures, and at the time of completing the self-reported measures—i.e., before the movement task—participants were unaware of the lifting task they had to perform. This prevented potential influence of movement exposure on the self-report or vice-versa. As such, the current study provides important additional evidence for the relationships between task-specific pain-related psychological variables and movement velocity and duration.

Despite the complex relationships between movement behaviour and pain-related psychological measures, the latter has typically been assessed using general self-report measures, such as the Tampa Scale for Kinesiophobia or the Fear Avoidance Beliefs Questionnaire. These general self-report measures do not assess beliefs about specific activities, but only contain items referring to physical activity or exercises in general. Yet, many patients with CLBP consider physical activity and exercises as beneficial [35], while they do fear and avoid specific activities (e.g., lifting with a bent back) [15]. This lack of specificity has been hypothesized as one of the main reasons why associations between these general self-report measures and movement behaviour are typically weak in LBP populations [16]. Various recent systematic reviews and meta-analyses have consistently reported these weak associations between general self-reported pain-related psychological measures and lumbar range of motion [12,13], muscle activity [12,13], physical performance measures (e.g., muscle strength) [11], and postural control [14]. Based on these findings and the emerging recent evidence—including the current study—showing that task-specific pain-related psychological measures better predict movement behaviour [16,17,18,19,20], a paradigm shift from using general to task-specific self-report measures is necessary to move the field forward in disentangling the intricate interactions between movement behaviour and pain-related beliefs.

Our study results may also have clinical implications. First, based on previous recommendations [36], clinicians might strongly rely on cut-off scores on general self-report measures of pain-related fear (e.g., TSK) to select patients for participating in exposure therapy in vivo, the recommended treatment for tackling avoidance behaviour and its related disability in CLBP [37]. However, given the lack of association between the TSK and (avoidant) movement behaviour, patients with low TSK-scores may still display avoidance behaviour. Consequently, using TSK cut-off scores as a selection criterion for participation in exposure therapy in vivo may prohibit those patients from being offered appropriate treatment. Second, it may be recommended to evaluate movement duration and velocity in patients with CLBP, as these are important parameters to characterize protective movement behaviour. Patients with CLBP often protect their lumbar spine by engaging in avoidance behaviour. Typically, this does not result in complete avoidance (e.g., not lifting objects at all), but rather in the adoption of so-called safety behaviours. These are behavioural adaptations that aim to prevent a feared outcome (e.g., only lifting in a slow and controlled manner as to avoid buckling of the lower back) [7]. From a clinical perspective, careful evaluation of these safety behaviours is essential, as they may need to be addressed during treatment, especially in patients where avoidance behaviour interferes with daily functioning [15,38]. More specifically, patients may need to be exposed to faster movements so they can experience and learn that these movements are safe to perform and that the feared outcome will not occur (e.g., the lower back will not buckle). This will result in extinction of the avoidance behaviour and enable patients to participate in valued life activities again or to perform them at more natural velocities, ultimately leading to reduced disability [39].

Various limitations apply to this study. First, we only used the TSK and its subscales as general measures of pain-related fear. Besides the TSK, also the Fear Avoidance Beliefs Questionnaire has been recommended to assess pain-related fear in LBP populations [40]. Although we cannot make any conclusions regarding the Fear Avoidance Beliefs Questionnaire, it is unlikely that its scores would be associated with the movement parameters assessed in this study. Meta-analyses have shown that correlation coefficients between the Fear Avoidance Beliefs Questionnaire—including its subscales—and various movement parameters are similar than such correlation coefficients using the TSK [11,12]. Second, we only used a lifting task to assess movement behaviour, so we should be prudent in extrapolating our findings to other tasks. Third, a significant proportion of the variability in movement behaviour remained unexplained, despite controlling for various parameters in the linear regression models. However, this should not come as a surprise, since movement behaviour can be influenced by a myriad of factors. For example, degenerative changes of the lumbar spine have been shown to affect lumbar range of motion [41], or changes in muscle activity patterns caused by acute LBP may persist in the chronic phase [33]. In this respect, it could have been useful to have measured muscle activity patterns to further explain the observed movement behaviour. Finally, although participants were instructed to lift in their habitual way, it cannot be excluded that contextual factors (e.g., being in a laboratory setting) may have influenced movement behaviour. Further exploration of movement behaviour in a more natural and relevant setting for the individual patient (e.g., at home) may provide additional insights into the relationships between movement behaviour and pain-related psychological measures.

## 5. Conclusions

Movement duration and movement velocity during a lifting task were best predicted by task-specific perceived harmfulness in patients with CLBP. Although the general measure of perceived harmfulness also predicted certain movement parameters, it explained clearly less variance than the task-specific measure of perceived harmfulness. General measures of pain-related fear were not predictive for any of the movement parameters. These results provide further evidence for using task-specific pain-related psychological measures for disentangling the relationships between pain-related beliefs and movement behaviour in patients with CLBP.

## Figures and Tables

**Table 1:** Sociodemographic data and baseline questionnaires.

	Pain-Free Persons (n = 54)	Patients with CLBP (n = 55)		*p*
Sociodemographic data				
Age (years)	36.9 (13.1)	41.1 (13.6)		0.11
Sex (n, %)	34 (62%)	26 (47%)		0.10
BMI (kg/m^2^)	23.2 (3.5)	24.2 (3.4)		0.13
LBP questionnaires		Mean (SD)	Median (IQR)	
LBP duration (years) ^a^		7.2 (7.3)	5 (2 to 11)	
NPRS now (0 to 10)		3.3 (2.1)	3 (2 to 5)	
NPRS 7 days (0 to 10)		4.6 (1.7)	5 (3 to 6)	
RMDQ (0 to 24) ^a^		8.0 (4.1)	7 (5 to 11)	
PHODA-Total (0 to 100)		41.0 (13.6)	42.1 (33.8 to 49.3)	
PHODA-Lift (0 to 100) ^a^		72.9 (18.9)	77 (60 to 89)	
TSK-Total (17 to 68)		36.5 (6.9)	34 (31 to 42)	
TSK-SF (5 to 20)		10.0 (3.2)	10 (7 to 12)	
TSK-AA (8 to 32) ^a^		17.5 (4.6)	16 (13 to 20)	

BMI = body mass index; CLBP = chronic low back pain; NPRS 7 days = average pain in the past 7 days; NPRS current = current LBP intensity; PHODA-Lift = score on the task-specific picture of the PHODA-SeV; PHODA-Total = total score on the PHODA-SeV; RMDQ: Roland Morris Disability Questionnaire; TSK-AA = score on the Activity Avoidance subscale of the TSK; TSK-SF = score on the Somatic Focus subscale of the TSK; TSK-Total = total score on the Tampa Scale for Kinesiophobia. ^a^ Data are not normally distributed.

**Table 2:** Correlation coefficients between pain-related psychological measures and movement parameters in patients with chronic low back pain (n = 55).

	LS (°/s)	L1 (°/s)	S1 (°/s)	Duration (s)
TSK-total	−0.11	0.05	0.17	0.02
	(0.43)	(0.71)	(0.23)	(0.86)
TSK-AA	−0.14	0.03	0.11	−0.02
	(0.30)	(0.85)	(0.41)	(0.88)
TSK-SF	−0.06	−0.03	0.09	0.01
	(0.69)	(0.84)	(0.51)	(0.92)
PHODA-Total	−0.13	−0.25	−0.18	0.37
	(0.36)	(0.07)	(0.19)	(0.007)
PHODA-Lift	−0.57	−0.55	−0.20	0.63
	(<0.0001)	(<0.0001)	(0.15)	(<0.0001)

LS = lumbar spine; PHODA-Lift = score on the task-specific picture of the PHODA-SeV; PHODA-Total: total score on the PHODA-SeV; TSK-AA = Activity Avoidance subscale of the TSK; TSK-SF = Somatic Focus subscale of the TSK; TSK = Tampa Scale for Kinesiophobia. Data are presented as correlation coefficients (*p*-value).

**Table 3:** Multiple linear regression models including the Total scores on the PHODA-SeV for predicting movement velocity and duration.

	Parameter	St. Beta	SE	*p*	R^2^ adj Basic Model	R^2^ adj Full Model	ΔR^2^ adj
LS velocity (°/s)	Sex	1.82	0.99	0.07	0.19	0.17	−0.02
	Age	−0.08	0.09	0.41			
	NPRS	−1.00	0.52	0.06			
	LBP duration	−0.01	0.15	0.95			
	RMDQ	−0.36	0.27	0.19			
	PHODA-Total	−0.03	0.08	0.71			
L1 velocity (°/s)	Sex	3.83	1.74	0.03	0.07	0.11	0.04
	Age	0.24	0.16	0.15			
	NPRS	−1.51	0.92	0.11			
	LBP duration	−0.30	0.19	0.11			
	RMDQ	−0.42	0.46	0.37			
	PHODA-Total	−0.23	0.13	0.09			
S1 velocity (°/s)	Sex	1.95	1.24	0.12	0.04	0.10	0.06
	Age	0.30	0.12	0.01			
	NPRS	−0.49	0.66	0.46			
	LBP duration	−0.25	0.13	0.06			
	RMDQ	−0.04	0.33	0.90			
	PHODA-Total	−0.20	0.09	0.04			
Duration (s)	Sex	0.044	0.030	0.15	0.03	0.11	0.08
	Age	0.001	0.003	0.67			
	NPRS	−0.012	0.016	0.47			
	LBP duration	0.004	0.003	0.26			
	RMDQ	0.002	0.008	0.76			
	PHODA-Total	0.005	0.002	0.02			
	Sex	0.053	0.025	0.04	0.03	0.37	0.34
	Age	0.002	0.002	0.45			
	NPRS	−0.011	0.013	0.40			
	LBP duration	0.004	0.003	0.20			
	RMDQ	−0.003	0.007	0.70			
	PHODA-Total	0.000	0.002	0.91			
	PHODA-Lift	0.007	0.002	<0.0001			

LBP duration = duration of the current LBP episode; LS = Lumbar spine; NPRS = Numeric Pain Rating Scale for current pain intensity; RMDQ = Roland Morris Disability Questionnaire; PHODA-Lift = scores on the task-specific measure of perceived harmfulness; PHODA-Total = total score on the PODA-SeV representing a general measure of perceived harmfulness. R^2^ adj Basic Model = the adjusted R^2^ of the multiple regression analysis only containing the control variables (sex, age, NPRS, Onset, and RMDQ). R^2^ adj Full Model = the adjusted R^2^ of the multiple regression analysis containing the basic model + the pain-related psychological variable. ΔR^2^ adj = the difference in adjusted R^2^ between the basic model and the full model, indicating the additional variance explained by adding the pain-related psychological measure to the basic model that only contains the controlling variables.

**Table 4:** Multiple linear regression models including the scores on the Task-specific measure of perceived harmfulness (PHODA-Lift) for predicting movement velocity and duration.

	Parameter	St. Beta	SE	*p*	R^2^ adj Basic Model	R^2^ adj Full Model	ΔR^2^ adj
LS velocity (°/s)	Sex	1.74	0.81	0.04	0.19	0.43	0.25
	Age	−0.10	0.07	0.20			
	NPRS	−0.95	0.43	0.03			
	LBP duration	0.00	0.12	0.99			
	RMDQ	−0.12	0.23	0.60			
	PHODA-Lift	−0.22	0.05	<0.0001			
L1 velocity (°/s)	Sex	3.43	1.57	0.03	0.07	0.26	0.19
	Age	0.21	0.15	0.16			
	NPRS	−1.53	0.83	0.07			
	LBP duration	−0.30	0.17	0.08			
	RMDQ	−0.19	0.42	0.65			
	PHODA-Lift	−0.32	0.09	0.0006			
S1 velocity (°/s)	Sex	1.64	1.26	0.20	0.04	0.06	0.02
	Age	0.29	0.12	0.02			
	NPRS	−0.56	0.67	0.41			
	LBP duration	−0.26	0.14	0.07			
	RMDQ	−0.06	0.34	0.85			
	PHODA-Lift	−0.11	0.07	0.13			
Duration (s)	Sex	0.053	0.025	0.04	0.03	0.38	0.35
	Age	0.002	0.002	0.45			
	NPRS	−0.011	0.013	0.40			
	LBP duration	0.004	0.003	0.19			
	RMDQ	−0.003	0.007	0.71			
	PHODA-Lift	0.007	0.001	<0.0001			

LBP duration = duration of the current LBP episode; LS = Lumbar spine; NPRS = Numeric Pain Rating Scale for current pain intensity; RMDQ = Roland Morris Disability Questionnaire; PHODA-Lift: Task-specific measure of perceived harmfulness. R^2^ adj Basic Model = the adjusted R^2^ of the multiple regression analysis only containing the control variables (sex, age, NPRS, Onset, and RMDQ). R^2^ adj Full Model = the adjusted R^2^ of the multiple regression analysis containing the basic model + the pain-related psychological variable. ΔR^2^ adj = the difference in adjusted R^2^ between the basic model and the full model, indicating the additional variance explained by adding the pain-related psychological measure to the basic model that only contains the controlling variables.

**Table 5:** Movement velocity and duration: comparison between the pain-free group and the complete CLBP group.

		Mean Estimate (SE)	Mean Difference (SE)	ES (*d*)	*p*
LS velocity (°/s)	Pain-free	28.1 (1.3)			
	CLBP	18.3 (1.3)	9.8 (1.8)	1.02	<0.0001
L1 velocity (°/s)	Pain-free	51.8 (2.0)			
	CLBP	39.6 (1.9)	12.2 (2.8)	0.85	<0.0001
S1 velocity (°/s)	Pain-free	23.2 (1.5)			
	CLBP	21.4 (1.4)	1.9 (2.1)	0.17	0.37
Duration (s)	Pain-free	1.20 (0.03)			
	CLBP	1.39 (0.03)	0.19 (0.04)	1.01	<0.0001

CLBP = chronic low back pain; ES = Cohen’s d effect size based on the difference with the pain-free group; LS = Lumbar spine.

**Table 6:** Movement velocity and duration: comparison between the pain-free group and CLBP subgroups based on the PHODA-Lift scores.

		Mean Estimate (SE)	Mean Difference (SE)	ES (*g*)	*p*
LS velocity (°/s)	Pain-free	28.1 (1.2)			
	Low	23.6 (2.2)	4.6 (2.5)	0.5	0.2
	Medium	18.8 (2.1)	9.4 (2.4)	1.05	0.0006
	High	13.3 (2.0)	14.8 (2.4)	1.69	<0.0001
L1 velocity (°/s)	Pain-free	51.8 (1.9)			
	Low	47.3 (3.4)	4.4 (3.8)	0.32	0.56
	Medium	42.3 (3.1)	9.4 (3.7)	0.7	0.03
	High	30.3 (3.1)	21.4 (3.6)	1.58	<0.0001
S1 velocity (°/s)	Pain-free	23.2 (1.5)			
	Low	23.7 (2.6)	−0.53 (3.0)	−0.05	0.99
	Medium	23.7 (2.4)	−0.53 (2.9)	−0.05	0.98
	High	16.9 (2.4)	6.2 (2.8)	0.59	0.08
Duration (s)	Pain-free	1.20 (0.02)			
	Low	1.24 0.04)	0.03 (0.05)	0.26	0.85
	Medium	1.37 (0.04)	0.17 (0.05)	1.13	0.001
	High	1.53 (0.04)	0.33 (0.04)	2.12	<0.0001

CLBP = chronic low back pain; ES = Hedges’ g effect size based on the difference with the pain-free group; LS = Lumbar spine. Mean scores (range) on the PHODA-Lift and number of participants per CLBP subgroup: Low (n = 18): 50.8 (range = 10–59); Medium (n = 18): 76.1 (range = 70–80); High (n = 19): 90.7 (range = 81–100).

**Table 7:** Movement velocity and duration: comparison between the pain-free group and CLBP subgroups based on the PHODA-Total scores.

		Mean Estimate (SE)	Mean Difference (SE)	ES (*g*)	*p*
LS velocity (°/s)	Pain-free	28.1 (1.2)			
	Low	19.4 (2.2)	8.8 (2.6)	0.93	0.003
	Medium	17.4 (2.2)	10.7 (2.6)	1.14	0.0002
	High	18.2 (2.3)	9.9 (2.7)	1.03	0.001
L1 velocity (°/s)	Pain-free	51.8 (2.0)			
	Low	44.4 (3.3)	7.5 (4.1)	0.54	0.16
	Medium	39.6 (3.4)	12.3 (3.9)	0.83	0.006
	High	34.7 (3.5)	17.1 (4.1)	1.15	0.0002
S1 velocity (°/s)	Pain-free	23.3 (1.5)			
	Low	25.1 (2.5)	−1.8 (2.9)	−0.16	0.59
	Medium	22.0 (2.5)	1.3 (2.9)	0.12	0.95
	High	16.7 (2.6)	6.5 (3.0)	0.59	0.09
Duration (s)	Pain-free	1.20 (0.03)			
	Low	1.31 (0.04)	0.10 (0.05)	0.53	0.11
	Medium	1.38 (0.04)	0.18 (0.05)	0.86	0.002
	High	1.48 (0.04)	0.28 (0.02)	1.34	<0.0001

CLBP = chronic low back pain; ES = Hedges’ g effect size; LS = Lumbar spine. Mean scores (range) on the PHODA-Total and number of participants per CLBP subgroup: Low (n = 18): 26.3 (range = 6.2–36.4); Medium (n = 18): 41.6 (range = 36.5–45.1); High (n = 19): 54.3 (range = 46.4–80.1).

## Data Availability

The datasets used and/or analysed during the current study are available from the corresponding author on reasonable request.

## Supplementary Materials

The following supporting information can be downloaded at: https://www.mdpi.com/article/10.3390/jcm13175025/s1, Table S1: Multiple linear regression models including the total scores on the Tampa Scale for Kinesiophobia for predicting movement velocity and duration; Table S2: Multiple linear regression models including the scores on the Activity Avoidance subscale of the Tampa Scale for Kinesiophobia for predicting movement velocity and duration; Table S3: Multiple linear regression models including the scores on the Somatic Focus subscale of the Tampa Scale for Kinesiophobia for predicting movement velocity and duration; Table S4: Movement velocity and duration: comparison between the pain-free group and CLBP subgroups based on the TSK-Total scores; Table S5: Movement velocity and duration: comparison between the pain-free group and CLBP subgroups based on the TSK-AA scores; Table S6: Movement velocity and duration: comparison between the pain-free group and CLBP subgroups based on the TSK-SF scores.
