# Supplementary material for: Task-Specific Perceived Harmfulness Predicts Protective Movement Behaviour in Chronic Low Back Pain
Source: J Clin Med. 2024 Aug 25;13(17):5025. doi: 10.3390/jcm13175025 (PMC11396003; doi:10.3390/jcm13175025)
Supplement: Supplementary file 1 [file jcm-13-05025-s001.zip › Table S1.pdf]

Table S1. Multiple linear regression models including the total scores on the Tampa Scale for Kinesiophobia for predicting movement velocity and duration

	parameter	St. Beta	SE	p	R^2 adj basic model	R^2 adj full model	ΔR^2 adj
LS velocity (°/s)	Sex	1.97	1.03	0.06	0.19	0.18	-0.01
	Age	-0.08	0.09	0.39			
	NPRS	-1.03	0.52	0.05			
	LBP duration	-0.01	0.14	0.95			
	RMDQ	-0.45	0.28	0.12			
	TSK-Total	0.10	0.16	0.55			
L1 velocity (°/s)	Sex	4.26	1.83	0.02	0.07	0.09	0.02
	Age	0.24	0.17	0.16			
	NPRS	-1.68	0.93	0.08			
	LBP duration	-0.31	0.19	0.11			
	RMDQ	-0.86	0.49	0.09			
	TSK-Total	0.40	0.29	0.17			
S1 velocity (°/s)	Sex	2.23	1.32	0.10	0.04	0.06	0.02
	Age	0.29	0.12	0.02			
	NPRS	-0.63	0.67	0.35			
	LBP duration	-0.26	0.14	0.06			
	RMDQ	-0.40	0.36	0.27			
	TSK-Total	0.30	0.21	0.15			
Duration (s)	Sex	0.055	0.033	0.10	0.03	0.01	-0.02
	Age	0.001	0.003	0.73			
	NPRS	-0.009	0.017	0.59			
	LBP duration	0.004	0.003	0.28			
	RMDQ	0.005	0.009	0.54			
	TSK-Total	0.002	0.005	0.75			

LBP duration= duration of the current LBP episode; LS= Lumbar spine; NPRS= Numeric Pain Rating Scale for current pain intensity; RMDQ= Roland-Morris Disability Questionnaire; TSK-Total: Total scores on the Tampa Scale for Kinesiophobia

R^2 adj basic model= the adjusted R^2 of the multiple regression analysis only containing the control variables (sex, age, NPRS, Onset and RMDQ)

R^2 adj full model= the adjusted R^2 of the multiple regression analysis containing the basic model + the pain-related psychological variable

ΔR^2 adj= the difference in adjusted R^2 between the basic model and the full model, indicating the additional variance explained by adding the pain-related psychological factor to the basic model that only contains the controlling variables.
