# Supplementary material for: Task-Specific Perceived Harmfulness Predicts Protective Movement Behaviour in Chronic Low Back Pain
Source: J Clin Med. 2024 Aug 25;13(17):5025. doi: 10.3390/jcm13175025 (PMC11396003; doi:10.3390/jcm13175025)
Supplement: Supplementary file 1 [file jcm-13-05025-s001.zip › Table S2.pdf]

Table S2. Multiple linear regression models including the scores on the Activity Avoidance subscale of the Tampa Scale for Kinesiophobia for predicting movement velocity and duration

	parameter	St. Beta	SE	p	R^2 adj basic model	R^2 adj full model	ΔR^2 adj
LS velocity (°/s)	Sex	1.98	1.01	0.05	0.19	0.18	-0.01
	Age	-0.08	0.09	0.37			
	NPRS	-1.05	0.52	0.05			
	LBP duration	0.00	0.14	0.97			
	RMDQ	-0.48	0.28	0.10			
	TSK-AA	0.20	0.24	0.40			
L1 velocity (°/s)	Sex	4.04	1.80	0.03	0.07	0.08	0.01
	Age	0.24	0.17	0.16			
	NPRS	-1.72	0.93	0.07			
	LBP duration	-0.30	0.19	0.11			
	RMDQ	-0.85	0.49	0.09			
	TSK-AA	0.53	0.41	0.20			
S1 velocity (°/s)	Sex	2.00	1.31	0.13	0.04	0.04	0.00
	Age	0.30	0.12	0.02			
	NPRS	-0.65	0.68	0.34			
	LBP duration	-0.26	0.14	0.07			
	RMDQ	-0.36	0.36	0.33			
	TSK-AA	0.33	0.30	0.27			
Duration (s)	Sex	0.057	0.032	0.08	0.03	0.02	-0.01
	Age	0.001	0.003	0.74			
	NPRS	-0.010	0.017	0.55			
	LBP duration	0.004	0.003	0.27			
	RMDQ	0.004	0.009	0.65			
	TSK-AA	0.005	0.007	0.46			

LBP duration= duration of the current LBP episode; LS= Lumbar spine; NPRS= Numeric Pain Rating Scale for current pain intensity; RMDQ= Roland-Morris Disability Questionnaire; TSK-AA: Scores on the Activity Avoidance subscale of the Tampa Scale for Kinesiophobia

R^2 adj basic model= the adjusted R^2 of the multiple regression analysis only containing the control variables (sex, age, NPRS, Onset and RMDQ)

R^2 adj full model= the adjusted R^2 of the multiple regression analysis containing the basic model + the pain-related psychological variable

ΔR^2 adj= the difference in adjusted R^2 between the basic model and the full model, indicating the additional variance explained by adding the pain-related psychological factor to the basic model that only contains the controlling variables.
