# Supplementary material for: Task-Specific Perceived Harmfulness Predicts Protective Movement Behaviour in Chronic Low Back Pain
Source: J Clin Med. 2024 Aug 25;13(17):5025. doi: 10.3390/jcm13175025 (PMC11396003; doi:10.3390/jcm13175025)
Supplement: Supplementary file 1 [file jcm-13-05025-s001.zip › Table S3.pdf]

Table S3. Multiple linear regression models including the scores on the Somatic Focus subscale of the Tampa Scale for Kinesiophobia for predicting movement velocity and duration

	parameter	St. Beta	SE	p	R^2 adj basic model	R^2 adj full model	ΔR^2 adj
LS velocity (°/s)	Sex	1.83	0.99	0.07	0.19	0.17	-0.02
	Age	-0.08	0.09	0.39			
	NPRS	-1.01	0.52	0.06			
	LBP duration	-0.02	0.14	0.87			
	RMDQ	-0.43	0.27	0.12			
	TSK-SF	0.17	0.32	0.59			
L1 velocity (°/s)	Sex	3.56	1.79	0.05	0.07	0.05	-0.02
	Age	0.24	0.17	0.16			
	NPRS	-1.63	0.95	0.09			
	LBP duration	-0.32	0.20	0.11			
	RMDQ	-0.63	0.48	0.20			
	TSK-SF	0.15	0.59	0.80			
S1 velocity (°/s)	Sex	1.65	1.30	0.21	0.04	0.02	-0.02
	Age	0.30	0.12	0.02			
	NPRS	-0.59	0.69	0.39			
	LBP duration	-0.25	0.14	0.08			
	RMDQ	-0.18	0.35	0.60			
	TSK-SF	-0.05	0.43	0.90			
Duration (s)	Sex	0.050	0.032	0.12	0.03	0.02	-0.01
	Age	0.001	0.003	0.70			
	NPRS	-0.009	0.017	0.59			
	LBP duration	0.004	0.003	0.24			
	RMDQ	0.008	0.009	0.35			
	TSK-SF	-0.006	0.010	0.57			

LBP duration= duration of the current LBP episode; LS= Lumbar spine; NPRS= Numeric Pain Rating Scale for current pain intensity; RMDQ= Roland-Morris Disability Questionnaire; TSK-SF: Scores on the Somatic Focus subscale of the Tampa Scale for Kinesiophobia

R^2 adj basic model= the adjusted R^2 of the multiple regression analysis only containing the control variables (sex, age, NPRS, Onset and RMDQ)

R^2 adj full model= the adjusted R^2 of the multiple regression analysis containing the basic model + the pain-related psychological variable

ΔR^2 adj= the difference in adjusted R^2 between the basic model and the full model, indicating the additional variance explained by adding the pain-related psychological factor to the basic model that only contains the controlling variables.
