# Supplementary material for: Task-Specific Perceived Harmfulness Predicts Protective Movement Behaviour in Chronic Low Back Pain
Source: J Clin Med. 2024 Aug 25;13(17):5025. doi: 10.3390/jcm13175025 (PMC11396003; doi:10.3390/jcm13175025)
Supplement: Supplementary file 1 [file jcm-13-05025-s001.zip › Table S4.pdf]

Table S4. Movement velocity and duration: comparison between the pain-free group and CLBP subgroups based on the TSK-Total scores

		Mean estimate (SE)	Mean Difference (SE)	ES (*g*)	p
LS velocity (°/s)	Pain-free	28.2 (1.3)			
	Low	18.8 (2.3)	9.4 (2.6)	0.97	0.001
	Medium	18.8 (2.1)	9.4 (2.5)	1.02	0.0009
	High	17.2 (2.3)	10.9 (2.7)	1.12	0.0003
L1 velocity (°/s)	Pain-free	51.8 (2.0)			
	Low	39.9 (3.5)	11.8 (4.0)	0.81	0.01
	Medium	39.6 (3.3)	12.2 (3.9)	0.85	0.006
	High	39.2 (3.5)	12.5 (4.2)	0.85	0.009
S1 velocity (°/s)	Pain-free	23.2 (1.5)			
	Low	21.1 (2.6)	2.1 (3.0)	0.19	0.84
	Medium	20.8 (2.4)	2.5 (2.9)	0.22	0.76
	High	22.3 (2.6)	0.9 (3.1)	0.08	0.98
Duration (s)	Pain-free	1.20 (0.03)			
	Low	1.34 (0.05)	0.14 (0.05)	0.64	0.02
	Medium	1.4 (0.04)	0.20 (0.05)	1.01	0.005
	High	1.42 (0.05)	0.22 (0.05)	1	0.003

CLBP= chronic low back pain; ES= Hedges' g effect size based on the difference with the pain-free group; LS= Lumbar spine.

Mean scores (range) on the TSK-AA and number of participants per CLBP subgroup: Low (n= 18): 26.6 (range= 23-32); Medium (n=18): 34.8 (range= 33-38); High (n=19): 44.7 (range= 40-53)
