# Supplementary material for: Task-Specific Perceived Harmfulness Predicts Protective Movement Behaviour in Chronic Low Back Pain
Source: J Clin Med. 2024 Aug 25;13(17):5025. doi: 10.3390/jcm13175025 (PMC11396003; doi:10.3390/jcm13175025)
Supplement: Supplementary file 1 [file jcm-13-05025-s001.zip › Table S5.pdf]

Table S5. Movement velocity and duration: comparison between the pain-free group and CLBP subgroups based on the TSK-AA scores

		Mean estimate (SE)	Mean Difference (SE)	ES (*g*)	p
LS velocity (°/s)	Pain-free	28.2 (1.3)			
	Low	19.2 (2.2)	9.0 (2.5)	0.95	0.002
	Medium	18.5 (2.2)	9.7 (2.6)	1.01	0.0009
	High	17.2 (2.3)	11.0 (2.7)	1.15	0.0003
L1 velocity (°/s)	Pain-free	51.8 (2.0)			
	Low	40.1 (3.4)	11.7 (3.9)	0.8	0.01
	Medium	39.8 (3.4)	12.0 (4.0)	0.82	0.009
	High	38..9 (3.6)	12.9 (4.1)	0.87	0.007
S1 velocity (°/s)	Pain-free	23.2 (1.5)			
	Low	20.8 (2.5)	2.5 (2.9)	0.22	0.77
	Medium	21.5 (2.5)	1.7 (3.0)	0.22	0.91
	High	21.8 (2.6)	1.4 (3.1)	0.13	0.96
Duration (s)	Pain-free	1.20 (0.03)			
	Low	1.38 (0.04)	0.18 (0.05)	0.86	0.002
	Medium	1.34 (0.04)	0.14 (0.05)	0.67	0.03
	High	1.45 (0.05)	0.24 (0.05)	1.15	<0.0001

CLBP= chronic low back pain; ES= Hedges' g effect size based on the difference with the pain-free group; LS= Lumbar spine.

Mean scores (range) on the TSK-AA and number of participants per CLBP subgroup: Low (n= 16): 11.8 (range= 9-13); Medium (n=20): 15.2 (range= 14-17); High (n=19): 22.2 (range= 18-27)
