# Supplementary material for: Task-Specific Perceived Harmfulness Predicts Protective Movement Behaviour in Chronic Low Back Pain
Source: J Clin Med. 2024 Aug 25;13(17):5025. doi: 10.3390/jcm13175025 (PMC11396003; doi:10.3390/jcm13175025)
Supplement: Supplementary file 1 [file jcm-13-05025-s001.zip › Table S6.pdf]

Table S6. Movement velocity and duration: comparison between the pain-free group and CLBP subgroups based on the TSK-SF scores

		Mean estimate (SE)	Mean Difference (SE)	ES (*g*)	p
LS velocity (°/s)	Pain-free	28.2 (1.3)			
	Low	18.3 (2.2)	9.9 (2.5)	1.04	0.0005
	Medium	18.8 (2.2)	9.4 (2.6)	0.99	0.002
	High	17.9 (2.3)	10.2 (2.7)	1.08	0.0007
L1 velocity (°/s)	Pain-free	51.8 (2.0)			
	Low	39.9 (2.0)	11.9 (3.9)	0.81	0.008
	Medium	40.4 (3.5)	11.5 (4.0)	0.77	0.01
	High	38.5 (3.5)	13.3 (4.1)	0.91	0.005
S1 velocity (°/s)	Pain-free	23.3 (1.5)			
	Low	21.5 (2.5)	1.8 (2.9)	0.16	0.89
	Medium	21.7 (2.6)	1.5 (3.0)	0.15	0.94
	High	20.8 (2.6)	2.5 (3.1)	0.23	0.79
Duration (s)	Pain-free	1.20 (0.03)			
	Low	1.38 (0.04)	0.18 (0.05)	0.86	0.002
	Medium	1.38 (0.04)	0.18 (0.05)	0.86	0.003
	High	1.40 (0.05)	0.20 (0.05)	0.92	0.001

CLBP= chronic low back pain; ES= Hedges' g effect size based on the difference with the pain-free group; LS= Lumbar spine.
Mean scores (range) on the TSK-SF and number of participants per CLBP subgroup: Low (n= 18): 6.4 (range= 5-8); Medium (n=18): 9.9 (range= 9-11); High (n=19): 13.4 (range= 12-18)
